# Supplementary material for: NPAS4 in the medial prefrontal cortex mediates chronic social defeat stress-induced anhedonia-like behavior and reductions in excitatory synapses
Source: eLife. 2023 Feb 13;12:e75631. doi: 10.7554/eLife.75631 (PMC9925055; doi:10.7554/eLife.75631)
Supplement: Transparent reporting form [file elife-75631-transrepform1.pdf]

1st Floor
24 Hills Road
Cambridge CB2 1JP, UK

P 01223 855340
W elifesciences.org
T @elife

weLife's transparent reporting form

We encourage authors to provide detailed information *within their submission* to facilitate the interpretation and replication of experiments. Authors can upload supporting documentation to indicate the use of appropriate reporting guidelines for health-related research (see EQUATOR Network), life science research (see the BioSharing Information Resource), or the ARRIVE guidelines for reporting work involving animal research. Where applicable, authors should refer to any relevant reporting standards documents in this form.

If you have any questions, please consult our Journal Policies and/or contact us: editorial@elifesciences.org.

Sample-size estimation

- You should state whether an appropriate sample size was computed when the study was being designed
- You should state the statistical method of sample size computation and any required assumptions
- If no explicit power analysis was used, you should describe how you decided what sample (replicate) size (number) to use

Please outline where this information can be found within the submission (e.g., sections or figure legends), or explain why this information doesn't apply to your submission:

We have used several cohorts of animals for multiple experimental purposes, including qPCR, immunohistochemistry dendritic spine quantification, and all behavioral analyses, except for RNA-sequencing data (two independent cohorts). No explicit power analysis was used prior to the study to determine sample sizes. Target sample sizes for behavioral analyses were based on previous publication using similar methods (Krishnan et al., 2007, Cell; Golden et al., 2011, Nat Protoc; Colyn et al., 2019, Behav Brain Res). For behavioral analysis on social interaction, we have found through an abundance of published data over the years that cohorts of at least 10 animals per group are required. The sample size for in-vivo experiments was also sufficient to get significant differences with the observed effect sizes. This information is mentioned in the Materials and Methods section and figure legends.

Replicates

- You should report how often each experiment was performed
- You should include a definition of biological versus technical replication
- The data obtained should be provided and sufficient information should be provided to indicate the number of independent biological and/or technical replicates
- If you encountered any outliers, you should describe how these were handled
- Criteria for exclusion/inclusion of data should be clearly stated
- High-throughput sequence data should be uploaded before submission, with a private link for reviewers provided (these are available from both GEO and ArrayExpress)

Please outline where this information can be found within the submission (e.g., sections or figure legends), or explain why this information doesn't apply to your submission:

The number of replicates for each experiment is stated in each figure legend or on the graphs themselves. Each animal is a biological replicate. All information is included in figure legends and in the materials and methods section. Outliers were identified using Prism 8 Identify Outliers function. If identified, the outliers were removed via Grubbs test (alpha 0.05).

Statistical reporting

- Statistical analysis methods should be described and justified
- Raw data should be presented in figures whenever informative to do so (typically when N per group is less than 10)
- For each experiment, you should identify the statistical tests used, exact values of N, definitions of center, methods of multiple test correction, and dispersion and precision measures (e.g., mean, median, SD, SEM, confidence intervals; and, for the major substantive results, a measure of effect size (e.g., Pearson's r, Cohen's d)
- Report exact p-values wherever possible alongside the summary statistics and 95% confidence intervals. These should be reported for all key questions and not only when the p-value is less than 0.05.

Please outline where this information can be found within the submission (e.g., sections or figure legends), or explain why this information doesn't apply to your submission:

Details of the statistical analyses are given in the main manuscript and figure legends. Each reference to a statistical test is accompanied by the sample size and appropriate metrics. Please also see the statistics table provided in a separate file attachment.

(For large datasets, or papers with a very large number of statistical tests, you may upload a single table file with tests, Ns, etc., with reference to sections in the manuscript.)

Group allocation

- Indicate how samples were allocated into experimental groups (in the case of clinical studies, please specify allocation to treatment method); if randomization was used, please also state if restricted randomization was applied
- Indicate if masking was used during group allocation, data collection and/or data analysis

Please outline where this information can be found within the submission (e.g., sections or figure legends), or explain why this information doesn't apply to your submission:

The animals were randomly selected to be used as control group or social defeat group upon arrival as described in the Method and Materials section.

Additional data files ("source data")

- We encourage you to upload relevant additional data files, such as numerical data that are represented as a graph in a figure, or as a summary table
- Where provided, these should be in the most useful format, and they can be uploaded as "Source data" files linked to a main figure or table
- Include model definition files including the full list of parameters used
- Include code used for data analysis (e.g., R, MatLab)
- Avoid stating that data files are "available upon request"

Please indicate the figures or tables for which source data files have been provided:

Source data for all figures have been provided. No code was used for data analysis. RNA-sequencing data are provided in a separate table. Please also see the provided table for reagents and primer sequences.

eLife Sciences Publications, Ltd is a limited liability non-profit non-stock corporation incorporated in the State of Delaware, USA, with company number 5030732, and is registered in the UK with company number FC030576 and branch number BR015634 at the address 1st Floor, 24 Hills Road, Cambridge CB2 1JP | August 2014
